# Not being heard: barriers to high quality unplanned hospital care during young people’s transition to adult services – evidence from ‘this sickle cell life’ research

**DOI:** 10.1186/s12913-019-4726-5

**Published:** 2019-11-21

**Authors:** Alicia Renedo, Sam Miles, Subarna Chakravorty, Andrea Leigh, Paul Telfer, John O. Warner, Cicely Marston

**Affiliations:** 1 London School of Hygiene and Tropical Medicine, London, UK; 2 King’s College Hospital NHS Trust, London, UK; 3 University College London Hospitals NHS Foundation Trust, London, UK; 4 Barts Health NHS Trust, London, UK; 5 Collaborations for Leadership in Applied Health Research and Care NW London, Imperial College London, London, UK

**Keywords:** Adolescents, Chronic illness, Community involvement, Long-term conditions, Patient involvement, Patient participation, People-centred healthcare, Transition, Sickle cell disease, Young people

## Abstract

**Background:**

Young people’s experiences of healthcare as they move into adult services can have a major impact on their health, and the transition period for young people with sickle cell disease (SCD) needs improvement. In this study, we explore how young people with SCD experience healthcare during this period of transition.

**Methods:**

We conducted a co-produced longitudinal qualitative study, including 80 interviews in 2016–2017 with young people with SCD aged 13–21 (mean age 16.6) across two cities in England. We recruited 48 participants (30 female, 18 male): 27 interviews were one-off, and 53 were repeated 2–3 times over approximately 18 months. We used an inductive analytical approach, combining elements of Grounded Theory and thematic analysis.

**Results:**

Participants reported significant problems with the care they received in A&E during painful episodes, and in hospital wards as inpatients during unplanned healthcare. They experienced delays in being given pain relief and their basic care needs were not always met. Participants said that non-specialist healthcare staff did not seem to know enough about SCD and when they tried to work with staff to improve care, staff often seemed not prepared to listen to them or act on what they said. Participants said they felt out of place in adult wards and uncomfortable with the differences in adult compared with paediatric wards. Because of their experiences, they tried to avoid being admitted to hospital, attempting to manage their painful episodes at home and accessing unplanned hospital care only as a last resort. By contrast, they did not report having problems within SCD specialist services during planned, routine care.

**Conclusions:**

Our study underscores the need for improvements to make services youth-friendly and youth-responsive, including training staff in SCD-specific care, compassionate care and communication skills that will help them elicit and act on young people’s voices to ensure they are involved in shaping their own healthcare. If young people are prevented from using transition skills (self-management, self-advocacy), or treated by staff who they worry do not have enough medical competency in their condition, they may well lose their trust in services, potentially compromising their own health.

## Background

Improving young people’s health and their transition to adult care is a global priority [1]. Health transition is a long process: it spans the period from early adolescence to adulthood and continues beyond transfer to adult care until the person is fully established as an adult patient in adult services [2] and represents a significant developmental phase [2, 3]. Transition is particularly important for people with chronic illness because it is a time of increased medical vulnerability [4] and increased responsibility for self-care that occurs while adult behaviours are developing and transitions are also occurring in other areas of life. Young people’s experiences of care during the overall process of transition, including the support they receive to adjust to adult services (i.e. “transitional care”) and their experiences of clinical care across the healthcare system during this period, can significantly affect their health and future life [5, 6]. Poor experiences of healthcare during this crucial stage of the life course are likely to influence their future patterns of interaction with services and may cause disengagement from services and poor health outcomes [7].

One chronic condition where there is an urgent need to improve the health transition process is sickle cell disease (SCD) [8]. SCD is a complex multisystem inherited blood condition characterised by episodes of acute and often severe pain, and progressive chronic organ damage which causes significant morbidity and mortality [9, 10]. A distinctive characteristic of SCD is the experience of unpredictable and episodic acute painful episodes that can be excruciating, caused by vaso-occlusion [11]. Acute pain requires timely treatment and for people with SCD is the most common reason for attendance at hospital emergency departments and hospitalisation [12, 13]. Acute pain episodes can be accompanied by other complications such as acute chest syndrome, acute stroke, and infections [11] which can have profound, long term health implications.

A defining characteristic of the experience of living with SCD is the uncertainty in everyday life caused by the unpredictability of the condition, including the ever-present threat of painful episodes and complications [14]. This means the period of transition to adult care – a period of increased vulnerability for children and young people with any chronic condition [15] – is likely to be particularly complex for those with SCD. In England, SCD mainly affects individuals from Black African and Afro-Caribbean groups [16] who are also more likely to suffer from health and social inequalities [17]. Complicating the situation further is the stigma that can be associated with the condition, ranging from its association with physical complications (e.g. jaundiced eyes) [18], to ‘drug-seeker’ stereotyping associated with the need for opioid-based analgesics [19], to the racialisation of SCD [20–22] and stigma associated with this diagnosis in some of the African communities where young people with SCD reside [23].

Failing to treat acute episodic pain adequately can lead to morbidity [24]. Yet a major problem for people with SCD is the delay they experience in receiving appropriate pain relief when they seek care in the UK [25], with young people aged 16–20 years reporting poorer experiences of care for SCD than other age groups, particularly when they seek emergency care [25]. The highest rates of emergency admission for SCD in England are among 20–29 year-olds [26], suggesting that young people reaching adulthood are not as well equipped as they should be to avoid emergency hospitalisations. There are also well-documented problems associated with the period of transition to adult care for young people with other health conditions [4, 27, 28] – SCD is unfortunately not unique.

Good transition care to prepare adolescents and young adults to adapt to adult services is increasingly considered a key element of quality for young-people-friendly healthcare services [6, 29]. While there is a considerable body of research examining how transition is managed in specialist care, there are some major gaps. For instance, young people’s voices are not yet sufficiently addressed in healthcare transition research ( [30], for exceptions see [31, 32]), and there is very little research examining how experiences of clinical care across the healthcare system (beyond specialised care) affect the transition process [33].

Understanding how young people view their experiences during the process of transition to adult care is crucial. Healthcare transition is complex [34, 35] and any efforts to support this process should consider the needs and experiences of young people across the healthcare system and during the post-transfer period [2, 36, 37]. Listening to children’s and young people’s voices - and acting on what they say - will help us develop better, more young-people-friendly services [2] and adolescent-responsive health systems [36], as well as improving quality of care [38].

In this study, we present young people’s experiences of healthcare during transition, focusing in particular on their experiences of clinical care outside the organised “transitional care” they receive to prepare them to move into adult services.

## Methods

Our project “This Sickle Cell Life” used a longitudinal qualitative design. The project was co-produced with SCD patient advocates from its inception through to analysis and dissemination [39]. Participants were 48 young people with sickle cell disease (30 women and 18 men aged 13–21).

We conducted 27 one-off interviews (17 with 19–21 year-olds, and 10 with 13–18 year-olds) and 53 repeated interviews with 21 13–18 year-olds, interviewing them 2–3 times over a period of approximately 18 months. Repeat interviews aimed to capture experiences in real time during transition to adult care. We conducted 80 interviews between January 2016 and September 2017 in London (the UK city where most people with sickle cell live [26]) and one other English city (to capture a wider range of experiences). AR, a social science researcher with a PhD in Social Psychology, employed by a university and with no connection to participants and to any health services or recruitment sites conducted the interviews.

We recruited young people via hospitals with specialised SCD services and from the wider community via our network of contacts with patient advocates. Participants were first approached in person or by phone by healthcare providers (when recruiting via services) and via phone by our patient advocate contact (when recruiting from the wider community outside services). At this stage participants were given information and the opportunity to be contacted by AR by phone if they were interested in participating. Sixteen people who AR approached by phone did not go on to participate: in most cases because they did not return phone calls or were too busy.

Interview topics were chosen in discussion with people with lived expertise of the condition either because they had sickle cell or cared for people with sickle cell (patient and carer experts) [39] (see Additional file 1). We discussed and tested interview questions with one young adult with SCD (patient expert on the project) who also participated in developing the questions and refining the topic guide (See [39] for more detail). We explored participants’ experiences of receiving healthcare and living with SCD as well as the changes they were experiencing in their life more generally, in areas such as education, relationships, life at home and work, and how these interrelated with their SCD. In repeated interviews we explored lived experiences and changes since the previous interview, as well as specific follow-up questions for individuals to revisit topics from the previous interview. Participants chose the interview locations, usually their homes but sometimes in hospitals. Only the participant and interviewer were present during interviews, which were 60–90 min long, audio-recorded, and transcribed verbatim by a transcription agency, with detailed checks for transcription accuracy by the research team. During interviews AR checked participant’s wellbeing, offering opportunities for breaks.

We analysed interviews using an inductive, iterative approach, combining some of the practical steps of Grounded Theory [40] and thematic analysis [41]. The coding frame (set of codes/analytic categories) used for the first analytical steps of categorising, synthesising and comparing text data [40] was developed inductively from the interview data set and was also based on our *a priori* interest in understanding young people’s experiences of interacting with healthcare services and receiving care (as reflected in the topic guide). The coding frame was also refined alongside data collection and analysis, including via discussions with the patient and carer experts involved in the project. During repeated rounds of coding and ‘memo-writing’ [40], and via reflective dialogues with service user representatives, we developed and refined analytical categories. During the analysis we drew on field notes taken after interviews and dialogues with user representatives to reflect on how we might have participated in the co-production of data and in shaping what participants chose to report and how they positioned themselves in the interview. We took into consideration how AR being a white, adult academic without SCD influenced interview dynamics. AR & CM explained to participants that we were independent researchers who did not work in healthcare service provision. We designed young-people-friendly research leaflets and tried to conduct interviews on participants’ ‘home turf’ to help underline that we were working from a university, not the healthcare system.

The study was approved by the London School of Hygiene & Tropical Medicine (Ref 10107) and NHS research ethics committees (REC 15/LO/1135). Participants aged 16–21 and parents/carers of 13–15 year olds gave informed consent to participate. Participants aged 13–15 additionally gave their informed assent. We provided referral information to participants for support on issues raised, and gave them high street gift vouchers to compensate them for their time. To protect anonymity, we use participant age range not exact age and only use a number-letter label to identify participants.

## Results

Participants reported smooth transitions into adult specialist care, but poor experiences with non-specialist, unplanned and emergency care. Specifically, they did not report any particular problems with routine haematology clinic appointments, either during transition to adult services, or once they were established in those services. However, they did report problems in general services which they had to attend when they needed unplanned care, such as in Accident and Emergency departments (A&E) or on general hospital wards. In the rest of this paper, we explore these problems.

### Barriers to receiving good, personalised, and responsive care: not being heard

#### Being denied timely and adequate pain relief

Young people reported that when they went to non-specialist services such as A&E departments and stayed in adult wards for acute painful episodes, they experienced delays in receiving pain relief and did not receive adequate doses or appropriate types of analgesia. They said non-specialist hospital staff would question or disregard their reports that they were in pain, or would not allow them to help decide how their pain should be managed. They told us of occasions when they had tried to explain to staff what method or amount of pain relief worked best for them, and had been ignored.

*You might have ringed [sic] the bell maybe about, I don’t know, five, six times and, er, while you’re rolling around, they’re [ward nurses] really busy, moving around helping other people and stuff. So you might have to hold on a bit, er, longer than you’d normally hold on. So you might still be in pain and then, er, they’d only come to you, you know, maybe two, three hours even after. And then of course they’d tend to you and by then it might, it, the pain might have risen so much that, you know, it’s, it’s, the painkillers won’t work so then you might have to use stronger painkillers […] If you hold the pain for too long, um, it gets worse than it was before (O4, 19-21 years old)*

Participants told us their requests for pain relief were not treated as urgent (O4) and that they felt “ignored” and “abandoned”. They said they were often asked to wait, were given excuses (e.g. staff being busy), or were questioned about the degree of pain they were experiencing. Participants talked to us about how important it is to act quickly during painful SCD episodes because of the extreme pain and other negative physical consequences of having to wait for pain relief; they told us that the stress of waiting also made the pain more acute and made it less likely that the subsequent dose of pain relief would work. Some participants perceived delays to be caused by staff being busy. Others, however, attributed delays to staff scepticism about their pain experiences. These participants told us they felt judged and misunderstood. Some said they felt that they were perceived to be liars or “drug addicts” (O1). I6 said he overheard nurses talking about his pain not being that bad (“he just wants some morphine”) but he did not complain about it, because “when you need help […] you just accept it”.

*It was a bad experience really [being on the ward] […] The doctors just didn't really believe… not believe, but they thought I was like, faking, or like doing it to get medication [analgesia] as if I, like, was addicted to medication. […] But I can be laughing but still in pain and that’s what people need to understand, like nurses and doctors and even people in general: that I can be in pain, anyone can be in pain and, like, still be laughing because we know how to… people, like kids or, you know, kids know how to deal with the pain and we just try to distract ourselves. (O1 13-15 years old)*

*When I was in… admitted […] they would like sometimes say, uh there’s nothing wrong with her, uh she’s lying about her illness […] It was difficult especially ‘cause I was in pain, like, I dunno… Why I would lie about something about coming to hospital? It doesn't even make sense. (Z1 16-18 years old)*

Participants said they felt that staff judged their pain relief needs based on how they looked (O1). They said staff did not listen to them when they said they were in pain and that this translated into being denied timely pain relief, not being admitted to hospital, or being discharged too early (Z1). Participants explained that they did not necessarily present typical signs of pain because they had learned how to control how they expressed pain, for instance to avoid worrying their relatives (who might be visiting the ward), because they wanted to avoid focusing on the pain, or because they would be embarrassed to cry or shout in front of strangers.

*I sort of got ignored [in the ward] when I was calm and everything was fine, and I needed my tablets to be a constant, like every four hours. […] I’d be left for about eight hours, and then suddenly I’d get like a really bad sickle attack […] And I was even more stressed out because no one was listening, and I’d go for all these hours without like any tablets [pain relief]. And then the doctor would react after or during, whilst I was having a really bad sickle attack, or I’d be like crying, or in the bed like hunched over. And that was the only time that she would listen, but when I was calm and OK, or just sat there and go OK, I need my tablets, right, I need it now, I’ve been tracking the times, and they’d be like OK, and then not come back. […] She’d only see when I was in pain, and then she’d react, and then it was too late. […] It went on for about five days […] the second I say I’m not fine, for doctors they don’t seem to always react (Z2 19-21 years old)*

Z2 told us how she tried to question staff about why they were not responding to her requests for pain relief but the situation did not improve so she decided to go home to self-manage pain there because she “couldn’t get better in their care”. Others told us they preferred to self-manage painful episodes and avoid hospital care, even when they knew they should go to hospital, and said this was because they had experienced poor management of pain relief on previous visits (O4).

#### Body management and basic care needs ignored

Participants said they were not listened to and not involved in decisions about how to manage their bodies. They disliked injections and did not like the ways that intravenous cannulas were inserted: often abruptly and in locations that they knew from past experience would not be successful. They said they would tell staff the best place to insert the cannula, but staff ignored them.

*At the [children’s ward] they put the needle erm here, then the vein collapsed and then they started going here. Erm, at [adult ward] they went back to here [to the collapsed vein] and the first few times I told them that it’s not gonna work and it was like they weren’t listening to me. […] They would still try and do it. […] And erm then it would fail, so it felt like they were putting a needle in for no reason. (U5 19-21 years old)*

Some participants talked about suffering from scars, heavy bleeding and pain after not being listened to and staff being too “rough”. One participant said she had had to remove a cannula herself because staff forgot to do so. I9 characterized doctors’ multiple attempts to give her an injection as being “stabbed” (see also Z1 below); she said she felt the doctors “didn’t even care”. When they were in pain or experiencing side effects from strong analgesics, it was even more difficult for young people to advocate for themselves, including communicating how they wanted their treatment to be managed. E1 told us about being “disorientated” because of the pain, and the shock and pain of having an arterial line being removed without having the stiches attached to it taken out (E1, 19–21 years old). A5, and Z1 told us:

*[Hospital staff] left a bit of it [midline in A5’s arm], like, hanging out for, um, it was because I was having [analgesia administered via an intravenous cannula] and fluids at the same time. And he left it hanging out so it was easier for the nurses. […] They [hospital staff] pushed it all the way in, to the point where it was uncomfortable and hurt a bit. Um, but when I told them, they wouldn’t really do anything about it. (A5 16-18 years old)*

*[…] there was also the blood clotting injection […] she said, “I don’t have time”, and then she just, just stabbed it in. […] The crisis was in my legs this time so I asked her, “can you take me to the toilet?” And she said, “I don’t have time, you have to ask another nurse.” (Z1 16-18 years old)*

Participants talked about lacking mobility and not always being treated with dignity during their stay on the ward. They explained the barriers they encountered when trying to overcome their lack of control over how their bodies were managed by staff. Two participants talked about being put on a bed far away from the toilet when they were unable to walk properly, and said that their requests for help to go to the toilet or for a commode were not treated as urgent (U2). Others talked about the frustration of being moved from ward to ward when in pain or having to wait for a hospital bed when resting is an essential part of managing painful episodes. They explained that they were moved either because staff could not decide whether they should be in an adult or paediatric ward (E5) or because there were no beds available. A5 told us that she might be moved to three different wards in a single hospital stay. This made her feel not “wanted there”. She also told us about nurses “ignor[ing]” that she was unable to walk even though she had brought her crutches and wheelchair from home. Instead nurses “went ahead and did things how they usually do it and did it their way”. U2 told us about her legs hurting and asking for the commode because she had a drip. She heard the nurse saying to the other nurse “all the time this one needs a commode, commode”, which made her feel “really upset”.

*It’s just sometimes, like, if I’m admitted, […] it’s, like, a bit of a, it’s like, really weird, because they’re like, you should [be] on the [adult ward] because you’re [16-18 years old], […] they’re [doctors] always getting confused […] It’s just, it’s kind of tiring. Especially if […I], have to walk around, or if I had, like, if I’ll be in a bed and I have to go somewhere different […] So […] I’ve been moved from a ward to another, and back again, then back to A&E, and then back to another ward. […] They don’t know where to… where I should be. So, it’s kind of like, here we go again […] And obviously I’ll be… there’s no one going to be there to fight my corner, because mum’s not going to be there. And so it’ll be like explaining to them, like: “no, this is what it is, this is what it is” […] So I want to try and, I want to try and stay, like, as healthy as I can. That way I don’t have to put myself in that, like, position. (E5 16-18 years old)*

These experiences had made E5 want to avoid hospital as much as possible. E5 had not changed her mind when we interviewed her again 6 and 12 months later. She reiterated how she was trying her “hardest” to “stay out of hospital” until she was 19 “because obviously they wouldn’t know where to put me”, calling this failure to provide her with care that met her needs “part and parcel of growing up”.

### Feeling out of place: unwelcoming adult wards

Participants talked about feeling out of place in the adult ward because of the characteristics of the space as well as the age difference between them and the other patients. The children’s ward was full of babies and they felt they had grown out of it yet navigating the adult ward had proven challenging. Participants drew on sensory language, describing the space as “grey”, “dark and gloomy”, smelling of “sanitiser and sadness” to characterise the adult ward as a “sad” and “boring” space versus the “colourful” and “bright” children’s ward. Participants talked about their first experiences in the adult ward; feeling “scared” because of being surrounded by adults and missing their parents, the disturbing sounds of patients screaming and shouting, the feelings of loneliness. Their first experiences in the adult ward included being adjacent to patients with acute illness, with delirium, or screaming at others.

*Seeing someone which is pretty old and is just near you by a few steps and you say, “OK he has cancer so OK it’s pretty sad this” […] it’s like the going from, from a happy and coloured [children] ward from a grey and sad ward. […] You’re just staying in the room [in the adult ward], like, in silence, doing nothing and thinking when you can go out or when you can go away from that room for, yeah, five minutes just to not think that the person next to you is going to die in about three days (U1 16-18 years old)*

E5, who characterised both the “people” and “atmosphere” in the adult ward as “grey”, told us how she had seen things there that “you can’t really unsee” and that get “ingrained in your brain”. Participants saw the adult ward as an unhealthy, sick space and talked about the sadness that comes from realising that patients around you are dying. Participants spoke about feeling lonely and bored in the adult ward. They highlighted the lack of entertainment. They explained that it was difficult to socialise, because they were not used to and felt uncomfortable interacting with adults, or because patients looked too ill. By contrast, participants talked about the children’s ward as a “happy” environment where they enjoyed socialising and liked the entertainment (e.g. DVD player) and ward activities such as the ward school.

Participants wanted to avoid the adult ward as much as possible. They preferred to stay at home and manage painful episodes there. They characterised home (and their own bedrooms) as the ideal space that would afford them the calmness needed for recovery. At home they had more “control” and felt more “independent” (I10) and “free” (Z1) to manage pain. They wanted to be around their “own” things and in their “own” bed. They had access to resources to help manage pain that they could not access on the ward such as hot water bottles, a hot bath, or a massage from a family member. They talked about the importance of having their family around checking up on them just in case they needed help. Having this resource was important because they wanted to avoid the uncertainty they had experienced in the past of not knowing whether nurses would respond to their requests for help or whether they would respond on time.

*In my room, I can just tune everything out and I find it easier to fall asleep, because I have my own bed, or like my own things around me. I know if I need to get a hot water bottle it’s just over there […] I can track my own medication, I can track my own intake [...] They don’t have those in hospitals [creams/oils to soothe the pain], and even if they did I’d have all those attachments on to me (Z2 19–21 years old).*

### Trying hard to stay out of hospital

Participants explained that they tried to “avoid” going to hospital during a painful episode as much as possible, and instead tried to “deal with it” by themselves (Z3), although they said that they knew this carried risks. The process of transitioning to adult services for our participants involved learning how to rely on themselves and self-manage pain episodes at home to avoid bad hospital experiences. This process also involved becoming aware of how non-specialist staff may lack knowledge about SCD care. Participants told us they had learned how to ration pain medications, and use self-soothing remedies such as self-massage, using ointments, or practising breathing techniques. They explained how they learned to differentiate different types of pain and assess what pain relief they needed. Participants said they only went to hospital if their self-management was no longer enough and they needed stronger analgesia.

*If I’m seriously ill, I’d come into hospital but if I’m ill and I feel like I can stay at home, I’ll stay at home […] If I need […] more pain management I’ll come here [hospital]. If not, then stay at home […] You just know in yourself really, like [when to go to hospital]. When I was younger I think I used to, er, be in hospital a lot more […] but now I’m older I can bear with it [pain] […] I felt like I’d rather stay at the other one [children’s ward] [clears throat] ‘Cause you don’t know ‘em. When, when you’re with like older people you don’t know, that person, like, could probably just die.* (Y4 19-21 years old)

*Only a few years ago, that’s when I was able to kind of cope better, and I had a better understanding of the kind of medicine I need, and if I need like the strongest out of the painkillers or just like a little bit. So I kind of developed and kind of grew on to how to take care of it myself. Until I reached this point, where I feel like I don’t need to go hospital sometimes, or sometimes I do, then... And as you grow older you’re going to be able to know the different types of pain also, like if it’s going to be a mild crisis, or if it’s going to be a really severe crisis. (Z1 16-18 years old)*

I9 (below) told us how she was avoiding going to hospital, and how she had started questioning whether she really needed to access care after an experience of having her pain ignored by A&E staff. She felt she had to “look iller” than she was, in order for her pain reports to be taken seriously.

*I always felt like it was kind of condescending [treatment from A&E doctors] and it was kind of like, yeah, you don’t really have this. You don’t fit the textbook definition, so you shouldn’t be here. As opposed to why are you feeling like this? How are you feeling? What could possibly be wrong? Let’s try and solve it. (I9 19-21 years old)*

Participants often mentioned they thought that non-specialist staff needed better basic general knowledge and awareness of SCD. Poor quality of care at A&E and during hospital admissions had led participants to mistrust non-specialist staff (O4 below). One participant (U9 19–21 years old) told us about an emergency visit to a new hospital: the nurse there had asked him how long he had had the SCD, seemingly unaware that it is a genetic condition. U9 said that after this experience, he avoided this hospital altogether, preferring to return to his original hospital for any routine treatment.

Participants reported having experienced mistakes in their treatment, and said that because of this they felt they had to double-check care practices to avoid being given overdoses or being given the wrong medicine. O1, for instance, told us about checking whether nurses were doing things “right” when being given medication and E1 said she called her mother from hospital because nurses were going to give her medications she had never heard of, and she wanted to check whether she could take them. One participant told us how he was attended by a paramedic who “didn’t know anything about it [SCD]” (U5 19–21 years old) and gave him too high a dose of morphine, which U5 had never had before. Participants often said staff should listen to their requests for pain relief and should learn about sickle cell: “I want them [nurses] to like, um, understand my condition, and if I, uh, if I’m in pain I should get some medicines.” (U2)

*I try my best to hold it [pain] at home, even though I know it, it’s, it’s the worst option, I shouldn’t be doing this, I should go straight in. […] There might not be any chairs in A&E, you might not even get like a, a bed to be on until they call you out […] The whole Emergency area, was a massive deal ‘cause that, that could also, um, make me decide if I want to go into hospital or not. And, um, yeah, and the wards of course. The wards might not be as helpful, as nice, so you might be a bit scared to go […] Sometimes you’re not too sure if the nurse who’s looking after you- you’re not too sure if they understand you enough. So they might give you some medications that you can’t have or they might give you medications that- um, you know, the wrong dose (O4 19-21 years old)*

From their experiences, participants had learned that they did not have a voice they could use to convey their SCD expertise and their knowledge of their own body when they interacted with non-specialist healthcare staff. They said they often found themselves having to repeat to staff how SCD affected them and what care practices were suitable or unsuitable. When they could not avoid a hospital admission, participants wanted to cut their stay as short as possible. Z2 talked about knowing what type of analgesia to ask for on the ward while recovering from an acute painful episode. She wanted to get out of hospital, and knew she did not want codeine because this would make her fall asleep, which would in turn prolong her stay. She “wanted to be able to control it” herself so she “requested Nurofen and paracetamol”.

## Discussion

Key challenges during transition to adult services for young people with sickle cell disease predominantly relate to problems connected with poor care during unscheduled hospital visits for acute painful episodes, and unplanned admissions onto general, non-specialist hospital wards. Our findings indicate that there are four main areas of concern; all related to the key domains of youth-friendly care that young people report as integral to their positive experiences of care [42].

First, young people do not always receive timely or adequate pain relief. Second, they are not always heard when they make requests relating to basic bodily care – something that was particularly problematic when the young person lacked mobility. When participants tried to work with healthcare staff to improve their own care, they said staff often responded in ways that suggested they were not listening or not prepared to act on what the young person said. Third, our participants found adult wards unwelcoming and wanted to avoid them. Fourth, during transition young people learn that non-specialist healthcare staff may not know enough about SCD, and may not always provide adequate medical care, and because of this, they try to avoid going to hospital for painful episodes. These problems are likely to be even more acute in hospitals which do not contain specialised SCD clinics because there will be no SCD specialists available to advocate for patients.

Our participants’ narratives suggest that the period of transitioning between paediatric and adult healthcare services, as part of broader transitions to adulthood, involves a process of realising the shortcomings of unplanned non-specialist hospital care. Our findings suggest that this period can involve developing mistrust in and disengagement from non-specialist hospital care, with young people learning that their condition can often be misunderstood by non-specialist staff, who will not always be responsive to their needs, and may not allow them to be involved in decisions about their care. Given these experiences, transitioning to adulthood, and into adult care, involves a push into self-reliance and into learning how to confine care to self-management within what young people – even knowing the risks involved – nevertheless consider to be a safer environment at home.

The idea that young people should take increased responsibility in managing their health is at the heart of transitional care policies and guidance (e.g. [29, 36]). This is taxing for young people with SCD, for whom the period of transition to adult healthcare services involves learning to access unplanned hospital care only as a last resort. Taking responsibility for self-management of pain episodes is important. Yet self-reliance is labour-intensive; it requires effort to self-manage pain at home to resist or delay hospital care for a SCD pain episode, knowing that doing so can be risky. Delaying hospital care can bring complications [43] and pain might be more difficult to treat [44]. Patterns of health promoting and health risk behaviours are often established during adolescence and young adulthood [45, 46], and so avoiding care and trying to rely on self-management may be carried through into adult life. This may indeed help explain why emergency admissions to hospital are highest in the 20–29 age group: this is the age where parents and carers perhaps have less influence and where avoiding hospital may be more possible for the many young people who have had bad experiences with unplanned care. While self-management is an important skill, for our participants it seemed to be something they were prematurely having to employ because of their worries about poor care, rather than a positive skill that they were learning as part of healthy transition to adulthood – an interpretation also supported by the poor health outcomes for people with sickle cell in this transitional age group.

Self-reliance requires effort to be vigilant about non-specialist hospital care while simultaneously managing a pain episode, perhaps while unable to speak, or under the effects of analgesia. The young person experiencing excruciating pain is confronted with the additional stress from lack of control over their treatment, and the uncertainty about whether their voice will be taken seriously and whether their care needs will be met. In this uncertain situation, high quality care relies on non-specialist staff being receptive, recognising the validity of the patient’s pain reports, and acting on them.

Our study suggests that experiences of uncertainty about care during the transition period add additional stress to what is already a very uncertain illness experience [14]. Sickle cell pain episodes are unpredictable, and may lead to complications and rapid deterioration. This makes it difficult for people with sickle cell to make plans [14]. Resorting to self-management of painful episodes, and delaying hospital care, may help young people feel that they can exert some control in this otherwise very uncertain landscape, because they do so despite knowing that delay to and avoidance of hospital treatment carries risks to their health.

While lack of timely provision of pain relief [25], lack of patient involvement in treatment decisions [19, 47] and poor understanding of SCD among providers in unscheduled care settings are wider issues for all sickle cell patients [19, 25], these might be worse during the transition period when patients have not yet been established in adult care, and when unaccompanied young people may not have developed advocacy skills to negotiate their right to quality care. Our findings support previous studies showing that young people’s pain reports are not always taken seriously by providers [48], despite the fact that pain management is a key indicator of quality care for young people with chronic illness [49]. Our study adds understanding of how these experiences turn healthcare transitions into a process through which young people learn how to avoid unplanned hospital care and rely on their own resources as much as possible to circumvent these barriers. The lack of voice experienced by young people generally in healthcare services [50, 51] is compounded in the case of the young patient with SCD [52] because not all service providers are familiar with its treatment [19, 48], because of stigma that can attach to the condition [18, 53] and because of stereotyping of patients as drug-seekers [47, 48, 54]. In other chronic conditions, some of our findings are also likely to apply. The lack of voice that young people experience in encounters with acute non-specialist healthcare staff, barriers to accessing timely and adequate medical care, and the ‘out-of-placeness’ they experience in adult wards may well be experienced more widely. Involvement in their own care, staff attitudes such as respect and openness to listen to patients’ concerns, and pain control, are issues raised by young people as central to the quality of their healthcare experience [42, 49, 55]. Yet lack of staff training on these issues is still a key barrier to delivering patient-centred care to adolescents and young people [56]. Recent guidance for the care of adolescents and young adults has been developed with recommendations to tackle these issues, including pain management, in acute healthcare settings [57] and across the whole healthcare system [56].

## Conclusions

Transitional care work often focuses on the individual capacities of young people, such as their confidence to move into adult services, self-management skills, and health literacy (e.g. [29, 34]),

We would argue that transitional care should be complemented with additional work beyond planned/specialist-led clinics to create safer care spaces for young people when they access unplanned care in general services. Empowering young people to take responsibility for their own health, to self-advocate for own needs and to negotiate services is not enough if we do not ensure that the clinical care they receive across the healthcare system is supportive of these skills. Our findings highlight the need to move beyond the individual patient with SCD to improve healthcare experiences during transition using a social-ecological approach that recognises the challenges young people encounter throughout their support system including during interactions with healthcare providers [48]. A sole focus on ‘improving’ individual behaviours without addressing the barriers they face risks alienating young people, and may even reinforce barriers to treatment that transitional care should address. For instance, a young person may have the necessary transition skills (self-management, health-related knowledge), but if they are treated in a service where they are prevented from using those skills, where they are ignored, or where they are not allowed to be involved in treatment decisions, they may well lose their confidence and trust in that service – and perhaps healthcare services more widely. Self-management should not be interpreted as leaving patients to handle problems on their own. It should be guided and supported by the healthcare team and include guidance for non-specialist health-care professionals to promote positive youth development [2].

Certain transitional care approaches in planned/specialist-led services such as joint paediatric/adult clinics and specific age-banded clinics work to improve the preparation young people need to transition to adult services [5, 32]. Our study underscores the importance of improvements at healthcare system level beyond transitional care to make healthcare services youth-friendly [42] and adolescent-responsive [36, 58]. This should include inpatient adolescent wards [56, 57] and developmentally appropriate care [2, 37], as well as training staff in transitional care [56, 59, 60], care for people with SCD [48], including pain management, and in compassionate care [57, 61] and communication skills such as how to elicit and listen to young people’s voices, to ensure that young people are involved in shaping their healthcare [56, 57]. Implementing the use of patient-held health and wellbeing passports, and taking on board the personal information these contain about patient care (including how to identify signs of pain) and patient likes and dislikes, may help staff deliver more responsive care, particularly in situations where the unaccompanied young person is unable to communicate [62]. Staff training should have an experiential learning component [60], perhaps using simulation [63], and which includes young people [56], to help improve attitudes towards young people [48] and ensure sensitivity to their changing biopsychosocial developmental needs across the healthcare system [2, 36, 56, 58]. Rather than focusing on “support[ing] young people in fitting in with the healthcare system” ([64]: 165), we need to emphasise how to engage with young people and adapt health services to meet their needs. Preparation of young people to move into adult services is important, but equally important are structural and organisational changes such as tailoring services to young people and, crucially, creating enabling spaces for young people to have a voice throughout the health system, not just within specialist services.

## Supplementary information

**Additional file 1.** Interview topic guides.

## Data Availability

The datasets generated and analysed for this study contain sensitive personal data that were collected from children and adolescents. These data will not be made freely available by depositing them in a publically available repository. However, we will accept legitimate requests to access the data; requests should be made to Professor Cicely Marston. All requests will be considered on a case by case basis.

## Abbreviations

A&E: Accident and Emergency department
NHS: National Health Service
SCD: Sickle cell disease
