# Supplementary material for: Not being heard: barriers to high quality unplanned hospital care during young people’s transition to adult services – evidence from ‘this sickle cell life’ research
Source: BMC Health Serv Res. 2019 Nov 21;19:876. doi: 10.1186/s12913-019-4726-5 (PMC6873494; doi:10.1186/s12913-019-4726-5)
Supplement: Supplementary file 1 — Additional file 1. Interview topic guides. [file 12913_2019_4726_MOESM1_ESM.docx]

### **Additional file 1: Interview topic guides**

The following are an outline of the interviews with examples of questions under each topic explored. They were used flexibly according to the responses of the interviewees and what had been covered in previous interviews (stage 1 & 2). We asked follow-up questions, which were in response to what interviewees were telling us. In some cases, some topics (and questions) were revisited and explored in more detail at stage 2 & 3 interviews.

**STAGE 1 INTERVIEW**

**Explanation of the interview**

This is an opportunity for me to learn about your life and what it means living with sickle cell. We will talk about your daily life, about your life at home, as well as your friends and your thoughts for the future. We are going to talk a bit about issues that young people like yourself are dealing with in their everyday lives, including socialising, friendships and relationships. We will also talk about what kind of care you receive now and expect to receive in the future.

It’s up to you what you want to talk about. I am interested in learning about your thoughts and experiences. There are no right or wrong answers and you can say as much or as little as you like. You don’t have to answer questions if you don’t want to.

**Learning about the person**

- Can you tell me a bit about yourself?
- How has sickle cell impacted on your life?
- How do you feel this is different from the daily life of other young people of your age who do not have sickle cell?

**Home experiences**

- Who do you live with?
- How do they help you? [e.g. clinic visit reminders, accompanying them to the clinic]

**Socializing, friendships and relationships**

- Do your friends know about your sickle cell? What were their reactions when you told them?
- Does having sickle cell make any difference to how you think about being in a relationship, either now or in the future?
- Can you tell me a bit more about some of your relationships? [if they are or were in a relationship] Does/Did your partner know that you have sickle cell?
- One of the things that often comes up in interviews is concerns about sex. How might SCD affect sex?
- Are there things to do with boyfriends and girlfriends you would say are different for young people with sickle cell?

**Managing SCD [explore for each context; educational and work]**

- How do you feel having sickle cell affects what you do at school/college/university/work?
- Is there anything you need to do at school/college/university/work to manage your condition?
- Does anyone at school/college/university/work know you have sickle cell? How did they find out?
- In your current school/college/university/work: How do you feel about communicating your needs to your teacher/employer?
- What about the other students/peers? How do you feel they understand how sickle cell affects you?
- Does your school/college/university/work have anything in place to support you?
- Are there any circumstances when you wouldn’t feel conformable going to school/college/university/work?

**Clinic and health services**

- How long have you been receiving care from [clinic]?
- What is like attending the clinic?
- Tell me about your last visit. How was it?
- [for those in adult services] What was it like moving to the adult clinic?
- [for those who have not been transferred] Currently you receive care at [clinic] clinic. Do you think the care you receive here will always continue to be like this? What do you think will happen in relation to the care you receive into the future? What would you like to happen to prepare you for transition?

**Transition to adulthood**

- I want to talk now a bit more about what it is like growing up and when someone becomes an adult.
- What do you think comes with being perceived as an adult by others?

**Future aspirations**

- We are going to finish soon but first I’d like to ask you about your hopes for the future.
- What would you like your life to look like in 10 years’ time?
- How do you think that you might be able to make these wishes a reality?

**Closing**

We’re nearly finished. Thank you for your time. I have really enjoyed talking to you and I’ve learnt a great deal from you. Talking to you is very helpful for our study. I would like to interview you again in 6 months time. Next time we meet we will be talking about similar issues but particularly about the things that have happened since last time I saw you. I look forward talking to you more. Was this interview how you expected it to be?

**STAGE 2 INTERVIEW**

*Incorporate specific questions for each participant arising from previous interview*

**Explanation of the interview**

Thank you for taking the time to meet with me again. Today the discussion will be similar to our last chat, in that I am interested in your experience and thoughts in relation to SCD.

This is an opportunity for me to learn about your life and what it means to live with sickle cell. We will talk about your daily life, about your life at home, as well as your friends and your thoughts for the future. We are going to talk a bit about issues that young people like yourself are dealing with in their everyday lives, including going out and relationships. We will also talk about what kind of care you receive now and expect to receive in the future.

It’s up to you what you want to talk about. I am interested in learning about your thoughts and experiences. There are no right or wrong answers, and you can say as much or as little as you like.

**Catch up on what has happened since our last interview**

- Tell me what’s been going on in your life since we last met.
- How has sickle cell impacted on your life since we last met?
- Tell me how has your life been at school/college/university/work with your sickle cell? [explore for each context]
- Have you been admitted to hospital since I last saw you? [explore experience in the ward and/or at A&E]. How could your experience improve?
- Have you been transferred to adult services? Tell me about your experience [explore differences between adult clinic versus children’s clinic and between adult ward versus children’s ward]

**Follow-up on particular issues from previous interviews**

- Events in their life that were discussed last time e.g. changes that have been coming up when we last spoke to them, including following up on their current health and treatment
- Future aspirations
- Last time we talked about some of the challenges that young people face as they grow up and become adults.
  - What do you see your next big change in your life will be?
  - What are your thoughts about the future? Any fears you might have?
  - What are your thoughts about leaving home?
  - How do you see SCD might impact you in the future?
  - How can your health services help you make this transition?

**Telling others**

- Let’s talk about how it feels telling others about your sickle cell.
- Who else knows about your sickle cell? At school? Amongst friends? Amongst family? At work?
- How did they find out?
- Do you remember how they responded? What was their reaction when you told them?
- Are you encouraged to talk about sickle cell outside home?
- Why is important for others to know you have sickle cell?
- Is there anyone you don’t really want to find out? Why? How will you go about trying to ensure they don’t know?

**Closing**

We’re nearly finished. Thank you for your time. I have really enjoyed talking to you and I’ve learnt a great deal from you. Talking to you is very helpful for our study. We will meet in 6 months time for the third and final time. Next time we meet we will be talking about similar issues but particularly about the things that have happened since last time I saw you. I look forward talking to you more. Was this interview like you expected it to be?

**STAGE 3 INTERVIEW**

*Incorporate specific questions for each participant arising from previous interview*

**Explanation of the interview**

Thank you for taking the time to meet with me again. This is the last time we will meet to have these interviews. I have really enjoyed listening to you and have learnt a great deal from you

**Catch up on what’s happened since last interview**

- Tell me what’s been going on in your life since we last met.
- How has sickle cell impacted on your life since we last met.
- Tell me how has your life been at school/college/university with your sickle cell? [explore for each context]

**Follow-up on particular issues from previous interviews**

- Events in their life that were discussed last time e.g. changes that were approaching when we last spoke to them, including following up on their current health and treatment

**Pain**

- Can you draw your pain or a pain crisis?
- Can you describe your drawing? Tell me what is happening here
- Can you describe me the pain?
- Now thinking about when you have pain/ a pain crisis, is there anything else that you need to draw?

**Fatigue**

- Some young people with sickle cell say they often feel tired. Does this happen to you? Tell me about it.
- How does it affect you?
- How is life at school/college/university/work when you are feeling tired? [explore for each context]
- How is it with friends when you are feeling tired?

**Managing SCD in everyday life**

- Can you draw a map of the places you go to as part of your everyday life (non-healthcare service spaces)
- Can you draw a map of the places you go to for healthcare services/as part of healthcare services use?
- Can you explain your drawing?
- Tell me how these spaces are related
- Tell me about your experience in these spaces.
- Tell me where do you feel most comfortable and why?
- Where do you go when you are in pain?
- Are there any places where you go to when you are in pain?
- Some young people with SCD say they need help. To what extent do you need others’ help?
  - To what extend do you feel independent?

**Future aspirations**

- Last time we talked about some of the challenges that young people face as they grow up and become adults.
- What do you see as your next big change in life?
- What are your thoughts about the future? Any fears you might have?
- What are your thoughts about leaving home?
- How do you see SCD might impact you in the future?
- How can services help you make this transition?

**Closing**

So, we are going to finish soon, but I’d like to just ask you about your hopes for the future. This study has been about trying to understand the lives of young people with sickle cell and the different challenges that they face as they are moving into adulthood (including adult services, college etc).

- What would you like us to tell others (parents, healthcare professionals, young people, teachers) to better understand the experience of young people with sickle cell?
- How can the needs of young people with sickle cell be better addressed?
- What do you think could be done to make it easier for young people growing up with sickle cell?
